# Gain-Framed Messages Do Not Motivate Sun Protection: A Meta-Analytic Review of Randomized Trials Comparing Gain-Framed and Loss-Framed Appeals for Promoting Skin Cancer Prevention

**DOI:** 10.3390/ijerph9062121

**Published:** 2012-06-05

**Authors:** Daniel J. O’Keefe, Daisy Wu

**Affiliations:** Department of Communication Studies, Northwestern University, 2040 Campus Drive, Evanston, IL 60208, USA; Email: YujingWu2014@u.northwestern.edu

**Keywords:** skin cancer prevention, message framing, gain-framed, loss-framed, persuasive messages, meta-analysis

## Abstract

Persuading people to undertake actions to prevent skin cancer is an important public health challenge. A number of studies have compared the effectiveness of gain-framed and loss-framed appeals in this domain, often expecting gain-framed appeals to be more persuasive. A meta-analytic review (*k* = 33, *N* = 4,168), however, finds no significant difference in the persuasiveness of gain- and loss-framed appeals for encouraging skin cancer prevention. This conclusion is unaffected by differences in the specific protective action advocated or by differences in the kind of outcomes invoked. But the results offer an intimation that men might be more susceptible to framing variations in this domain—with loss-framed appeals potentially having a persuasive advantage.

## 1. Introduction

Skin cancer is a serious disease. One estimate is that almost 200,000 people worldwide were diagnosed with malignant melanoma in 2008 [1]. In the United States alone, the American Cancer Society’s estimate is that 12,190 deaths (9,180 from melanoma and 3,010 from other nonepithelial skin cancers) will occur in 2012 [2]. CDC’s National Program of Cancer Registries listed the skin as one of the top ten cancer sites in 2008 in the U.S. population [3]. The total number of people in the US population with nonmelanoma skin cancer has been estimated to have been over 3.5 million in 2006, making it an “underrecognized epidemic” [4].

It is correspondingly important to identify what sorts of persuasive messages might be most effective in encouraging skin cancer prevention. One choice faced by advocates is whether to use a gain-framed appeal, which emphasizes the desirable consequences of undertaking the recommended preventive actions, or a loss-framed appeal, which emphasizes the undesirable consequences of failing to undertake the recommended actions. 

The most well-known hypothesis about message framing persuasive effects is that the relative persuasiveness of gain-framed and loss-framed messages will vary depending on the kind of behavior being advocated. Specifically, the expectation has been that loss-framed appeals will be more persuasive than gain-framed appeals where the advocated action is a disease detection behavior, but gain-framed appeals will be more persuasive than loss-framed appeals where the advocated action is a disease prevention behavior [5,6]. This general hypothesis has not fared well, however. For most disease detection behaviors, there is no difference in the persuasiveness of gain-framed and loss-framed messages [7]. Similarly, for most disease prevention behaviors, there is no difference in the persuasiveness of gain-framed and loss-framed messages [8]. However, even if these broad categories of behavior do not yield the expected differences, those differences might still emerge in some more specific behavioral domain, such as skin cancer prevention.

This paper provides a meta-analytic review of the accumulated research concerning the relative persuasiveness of gain-framed and loss-framed messages for encouraging skin cancer preventive behaviors. The primary research question is whether gain-framed and loss-framed appeals are differentially persuasive on this subject. Plainly, if one appeal form is generally more effective than the other, those charged with designing messages on this topic will be able to craft messages in ways that maximize effectiveness. No previous message framing meta-analysis located more than a dozen studies concerning skin cancer prevention [8,9,10]. However, as will be seen, over 30 relevant studies are available (over twice as many as ever previously reviewed), which means that any previous conclusions would have been based on only a fraction of the relevant research evidence.

In addition to addressing the question of whether there is a general difference in the persuasiveness of gain-framed and loss-framed messages in this domain, we were also interested in exploring the possible moderating roles of three variables. One was the advocated action. Any number of different behaviors might be encouraged as a means of reducing skin cancer risk: using sunscreen, avoiding sun exposure at peak times, wearing protective clothing, and so forth. It might be that the relative persuasiveness of gain- and loss-framed appeals varies across different advocated actions.

A second was the basis of the persuasive appeal, that is, the kind of outcomes invoked by the appeal. Skin cancer preventive behaviors might be encouraged by emphasizing the dangers of skin cancer (or other health-related consequences)—or those behaviors might be encouraged by invoking appearance-related consequences of unprotected sun exposure, such as unattractive skin. It might be that the relative persuasiveness of gain- and loss-framed appeals varies depending on the basis of the appeal. 

A third was the sex of message recipients. Even though gain- and loss-framed appeals do not generally differ in persuasiveness, a small but statistically significant loss-framed advantage has been reported for studies in which messages advocated breast cancer detection behaviors [7]. This advocacy subject might be thought distinctive on any number of grounds, but given that all of the research participants in those studies were women, it is at least possible that men and women differ in their susceptibility to message framing variations. 

## 2. Methods

### 2.1. Identification of Relevant Investigations

#### 2.1.1. Literature Search

Research reports were located through personal knowledge of the literature, examination of reference lists from previous reviews and primary research reports, and searches of relevant databases (including Medline, PsycINFO, EBSCO, ProQuest Dissertations and Theses, ABI/INFORM, and Web of Science) using appropriate combinations of search terms such as *message framing*, *gain-framed*, *loss-framed*, *skin cancer*, *sunscreen*, and *sun avoidance*. 

#### 2.1.2. Inclusion Criteria

Studies selected had to meet three criteria. First, the study had to be a randomized trial comparing the persuasiveness of gain-framed and loss-framed messages. A gain-framed message emphasizes the advantages of compliance with the advocated view; a loss-framed message emphasizes the disadvantages of noncompliance. Second, the messages had to advocate skin cancer preventive behaviors (e.g., sunscreen use, minimization of sun exposure, *etc.*). Third, appropriate quantitative data relevant to persuasive effects (e.g., attitude, intention, or behavior) had to be available; where it was not provided in the report, we made efforts to obtain information from authors. Excluded by these criteria were studies of other kinds of framing variations (e.g., [11,12]), studies lacking an appropriate outcome variable [13], and studies for which relevant quantitative information could not be obtained [14,15,16,17,18].

### 2.2. Outcome Variable and Effect Size Measure

The outcome variable was persuasion, as assessed through attitude, behavioral intention, behavior, and the like. When multiple indices of persuasion (e.g., assessments of attitude and intention) were available, we averaged the effects to yield a single summary. Every comparison between a gain-framed message and its loss-framed counterpart was summarized using *r* (correlation) as the effect size measure. Differences indicating greater effectiveness with gain-framed messages were given a positive sign. When correlations were averaged (e.g., across several indices of persuasiveness), we computed the average using the *r*-to-*z*-to-*r* transformation procedure, weighted by *n*. 

### 2.3. Moderator Variables

#### 2.3.1. Advocated Behavior

We distinguished studies based on the behavior being recommended by the message. In nearly all studies, the messages mentioned sunscreen use, but some messages advocated only sunscreen use. Hence we contrasted studies in which the messages advocated sunscreen use and studies in which in the messages advocated either some other protective action (e.g., sun avoidance) or multiple actions (e.g., both sunscreen and sun avoidance). 

#### 2.3.2. Appeal Basis

We distinguished studies based on the kinds of outcomes that formed the basis of the persuasive appeals. In nearly all studies, the messages mentioned health-related outcomes, but some messages mentioned only health-related consequences. Hence we contrasted studies in which the messages invoked only health-related outcomes (e.g., skin cancer) and studies in which the messages did not invoke only health-related outcomes (appeals that referred to other outcomes such as appearance-related effects, or appeals that invoked a combination of health-related outcomes and other outcomes). 

#### 2.3.3. Message Recipient Sex

For each study for which appropriate exact information was available, we recorded the proportion of female participants. 

### 2.4. Unit of Analysis and Meta-Analytic Procedures

The unit of analysis was the message pair, that is, the pair composed of a gain-framed message and its loss-framed counterpart. We recorded a measure of effect size for each distinguishable message pair found in the body of studies. Whenever a study included more than one message pair and reported data separately for each pair, each pair was treated as providing a separate effect size estimate (e.g., [19]). 

In some cases, the same primary data served as the basis for multiple reports (e.g., both a dissertation and a subsequent publication). When a given investigation was reported in more than one outlet, it was treated as a single study and analyzed accordingly. The same research was reported (in whole or in part) in: [20] and ([21], Study 2); [22] and [23]; [24], [25], and [26]; [27] and [28]; [29] and [30]; [31] and [32]; [33] and [34]; [35] and [36]; [37] and [38]. The individual effect sizes (correlations) were analyzed using random-effects procedures (specifically, those of [39]).

## 3. Results

### 3.1. Sample Characteristics

Precise data were not available for most cases concerning the racial/ethnic composition of the study samples, although when such data were reported the samples were predominantly Caucasian. Most participants were young adults (e.g., university students).

### 3.2. Overall Effects

Effect sizes were available for 33 cases, with 4,168 participants. Details for each included case are contained in Table 1. Across all 33 cases, the random-effects weighted mean correlation was −0.020. The limits of the 95% confidence interval for this mean were −0.060 and 0.019, indicating no significant persuasive advantage for one framing form over the other (*Z* = −1.002, *p* = 0.316). 

**Table 1:** List of cases.

Study	*r*	*N*	Codings ^a^
Block (1993) sun exposure [20]	0.174	58	2/1/na
Cox, Cox, & Zimet (2006) Study 1 prevention [40]	0.013	139	2/1/41
Currie (2010) [41]	0.064	131	1/2/73
Detweiler *et al.* (1999) [42]	0.122	217	2/2/76
Fischer & Nabi (2001) sunscreen [43]	−0.191	79	1/1/54
Hoffner & Ye (2009) [28]	0.000	154	1/1/66
Hwang, Cho, Sands, & Jeong (in press) [23]	−0.099	219	2/2/45
Ku (2008) skin cancer [44]	−0.155	467	2/1/45
Lee & Aaker (2004) Experiment 2 promotion [45]	0.055	85	1/2/na
Lee & Aaker (2004) Experiment 2 prevention [45]	−0.173	78	1/2/na
Lee, Brown, & Blood (2000) sunscreen/clothing [46]	0.119	132	2/1/na
Lemieux, Hale, & Mongeau (1994) vivid high fear [19]	0.039	51	2/1/na
Lemieux *et al.* (1994) pallid high fear [19]	0.132	50	2/1/na
Lemieux *et al.* (1994) vivid low fear [19]	0.070	50	2/2/na
Lemieux *et al.* (1994) pallid low fear [19]	0.019	50	2/2/na
McCormick (2010) [47]	−0.114	154	1/2/na
Nan (2011) [30]	0.059	152	2/2/na
Robinson (2004) Study 1 [48]	−0.013	96	1/2/100
Rothman *et al.* (1993) Experiment 2 [18]	0.039	108	2/1/na
Saadi (2009) Experiment 1 prevention disincentive [49]	0.183	50	1/2/na
Saadi (2009) Experiment 1 prevention incentive [49]	−0.137	50	1/2/na
Schubert (2008) [50]	−0.131	174	2/2/na
Seo (2008) verbal [31]	0.191	48	1/2/na
Seo (2008) visual [31]	−0.162	47	1/2/na
Shamaskin (2009) Study 1 skin cancer prevention [33]	−0.165	49	2/1/59
Shen (2005) Study 1 skin cancer [35]	−0.054	286	2/2/73
Shen & Kollar (2011) sunscreen [51]	0.125	148	1/2/na
Shen & Kollar (2011) tanning [51]	−0.051	143	2/2/na
Smith (2003) prevention [52]	−0.008	31	2/2/na
Stoner (2010) [37]	0.110	136	2/1/100
Taber (2010) [53]	−0.101	146	2/1/60
Thomas *et al.* (2010) appearance [26]	−0.079	183	2/2/na
Thomas *et al.* (2010) health [26]	−0.144	207	2/1/na

**^a ^**The codings are, respectively, the advocated behavior (1 = sunscreen, 2 = other/multiple), the basis of the appeals (1 = health consequences only, 2 = other/multiple), and the proportion of female participants (na = not available).

This result was based on combining outcome data across attitudinal, intention, and behavioral outcomes—thereby affording greater statistical power for detecting any effects—but the substantive conclusion is unaffected by this procedural decision. In this dataset, as in other meta-analytic reviews of the relative persuasiveness of gain-loss message framing variations (e.g., [9,10]), these different outcomes were functionally equivalent as indicators of relative persuasiveness. Specifically: The mean ES for attitudinal outcomes (−0.017, *k* = 18, 95% CI limits of −0.078 and .044) did not significantly differ from the mean ES for intention outcomes (−0.013, *k* = 30, 95% CI limits of −0.055, 0.029; Q(1) = 0.010, *p* = 0.921) or from the mean ES for behavioral outcomes (0.044, *k* = 3, 95% CI limits of −0.140 and 0.225; Q(1) = 0.371, *p* = 0.543). The mean ES for intention outcomes did not significantly differ from the mean ES for behavioral outcomes (Q(1) = 0.344, *p* = 0.558).

### 3.3. Moderating Factors

#### 3.3.1. Advocated Behavior

The relative persuasiveness of gain-framed and loss-framed appeals was not affected by whether the messages advocated only sunscreen use (mean *r* = −0.013) or advocated other or multiple behaviors (mean *r* = −0.023); these two mean effects were not significantly different (*Q*(1) = 0.058, *p* = 0.810). 

#### 3.3.2. Appeal Basis

The relative persuasiveness of gain-framed and loss-framed appeals was not affected by whether the messages invoked only health-related consequences (mean *r* = −0.024) or did not invoke only health-related consequences (*i.e.*, invoked non-health consequences or a combination of health and non-health consequences; mean *r* = −0.015); these two mean effects were not significantly different (*Q*(1) = 0.048, *p* = 0.826). 

#### 3.3.3. Message Recipient Sex

Twelve cases provided exact information about the proportion of female participants. For exploring the possible moderating role of participant sex, we analyzed the data in two ways. First, we performed a modified weighted least squares regression using the proportion of female participants (as a continuous variable) to predict effect size. Using SPSS regression analysis, and applying the corrections described by [54], the proportion of female participants was strongly (*R* = 0.635) and significantly (*z* = 3.00, *p* = 0.001) related to effect size: As the proportion of female participants increased, effect sizes became more positive.

Second, we dichotomized the 12 relevant cases on the basis of the proportion of female participants. As indicated in Table 2, when the proportion of female participants was greater than 0.6, there was no significant difference in the persuasiveness of gain-framed and loss-framed appeals (mean *r* = 0.033, *k* = 6), but when the proportion of female participants was 0.6 or less, loss-framed appeals were significantly more persuasive than gain-framed appeals (mean *r* = −0.119, *k* = 6). These two mean effects were significantly different (*Q*(1) = 13.194, *p* = 0.001).

**Table 2:** Summary of results.

	*k*	*N*	mean *r*	95% CI	Power ^a^	*Q*(df)
All cases	33	4,168	−0.020	−0.060, 0.019	0.99	47.8(32) *
Advocated behavior						
sunscreen	12	1,120	−0.013	−0.085, 0.060	0.64	15.4(11)
other/multiple	21	3,048	−0.023	−0.071, 0.025	0.97	31.8(20) *
Appeal basis						
health	13	1,776	−0.024	−0.094, 0.046	0.84	23.2(12) *
other/multiple	20	2,392	−0.015	−0.061, 0.032	0.93	23.5(19)
Proportion of females						
>60%	6	1,020	0.033	−0.030, 0.096	0.61	5.1(5)
≤60%	6	1,099	−0.119	−0.177, −0.060	--	3.7(5)

* *p* < 0.05. **^a ^**These are power figures for detecting a population effect size of *r* = ±0.10, assuming large heterogeneity, with a random-effects analysis, .05 alpha, and a two-tailed test [55].

## 4. Discussion

Gain-framed and loss-framed appeals do not generally differ significantly in persuasiveness concerning skin cancer prevention. The lack of a statistically significant difference is not the consequence of poor statistical power. On the contrary, for detecting even a relatively small population effect (*r* = ±0.10), the data in hand afforded power of 0.99. One can be quite sure that if any nonzero population effect exists, it is quite small indeed. 

This is unquestionably a disappointing result, because identifying dependable ways of substantially enhancing the persuasiveness of skin cancer prevention messages is an important enterprise. But it is plain that those charged with the task of designing skin cancer prevention messages should generally not spend time worrying about whether their messages are gain-framed or loss-framed. 

This conclusion is unaffected by the specific preventive action being recommended. There is no evidence that the relative persuasiveness of gain-framed and loss-framed appeals varies as a consequence of whether the message advocates simply sunscreen use or advocates some other action or combination of actions. And this conclusion is unaffected by the kinds of consequences invoked by the message’s arguments. Specifically, there is no reason to suppose that the relative persuasiveness of gain-framed and loss-framed messages varies as a consequence of whether the messages invoke only health-related consequences (such as skin cancer) or invoke other consequences (in addition to, or instead of, health consequences). 

However, these negative conclusions must be tempered a bit by the intriguing result concerning the sex of message recipients. The very limited evidence in hand suggests that as the proportion of male message recipients increases, loss-framed appeals become progressively more advantageous than gain-framed appeals. More specifically, when the audience is predominantly female, message framing variations make virtually no difference—but with a larger proportion of men in the audience, a difference between gain- and loss-framed messages emerges, with loss-framed appeals having a persuasive advantage. 

It is not obvious what might explain this apparent effect. The effect is not plausibly a matter of men somehow being intrinsically more susceptible to message framing variations than are women. As mentioned above, a small but statistically significant difference between gain- and loss-framed appeals has appeared in studies in which all the participants were women (studies concerning breast cancer screening behaviors) [5]. It may simply be that this result is a statistical fluke; one notices, for example, the relatively poor statistical power in the set of studies in which the proportion of female participants exceeded 0.6. 

This explanatory uncertainty may be seen to reflect the key limitation of the present review (a limitation common to meta-analytic reviews), namely, that the conclusions are necessarily circumscribed by the nature of the primary research under investigation. Given that the observed sex-related effect may be a statistical oddity (given the relatively low power), only additional primary research can provide clarification. As another example: Nearly all of the samples in these studies were composed of college-age participants. Although this population is of natural interest as a focus for skin cancer prevention efforts, this restriction means that one cannot say with confidence how the results might have been different had a broader age range been sampled.

## 5. Conclusions

These results suggest three broad conclusions. First, gain-framed and loss-framed appeals do not generally differ in persuasiveness concerning skin cancer prevention. Researchers interested in developing understandings of what makes for more and less effective messages in this domain should consider directing their efforts elsewhere. 

Second, researchers who do continue testing potential differences in the persuasiveness of gain-framed and loss-framed messages concerning skin cancer prevention might attend closely to the sex composition of their samples. The present results raise the possibility of sex-related variations in the differential persuasiveness of gain- and loss-framed appeals, so further evidence will be welcomed.

Third, replications are essential in studies of the relative effectiveness of alternative public health messages. One of the earliest studies of message framing and skin cancer prevention—the research of Detweiler *et al.* [42]—compared one gain-framed brochure and one loss-framed brochure, and reported that the gain-framed message was more effective. This experimental design is the conventional one for addressing research questions concerning the relative effectiveness of two message kinds: one specific concrete message of one type is compared to one specific concrete message of the other type. But the result obtained in this study was obviously not general. Across 33 such comparisons to date, gain-framed and loss-framed messages are actually statistically indistinguishable with respect to persuasiveness concerning skin cancer prevention. This pattern of effects—in which an initial study produces a statistically significant effect but subsequent research fails to confirm it—is not uncommon [56,57]. Thus in addition to serving as a reminder to be cautious about the conclusions to be drawn from any single study, the present results underscore the importance of replications.

However, it should be noticed that where the research question concerns the relative persuasiveness of two message kinds (e.g., gain-framed *vs.* loss-framed messages), replications can be created within the design of a single experiment. That is, rather than conducting an experiment examining just one pair of messages (as is the current practice), a researcher could instead compare multiple message pairs. Such designs require statistical analyses adapted to the presence of message replications, but obviously have substantial benefits with respect to establishing dependable generalizations about message effects [58,59,60]. Thus in future research aimed at examining the relative effectiveness of two message kinds for encouraging skin cancer prevention (or any other behavior), the research community need not wait for replications to be published; researchers can-and arguably should-be asked to include message replications in their designs, so as to provide evidence that any claimed effects generalize beyond that particular pair of messages. 
